# Accommodative spasm in siblings: A unique finding

**DOI:** 10.4103/0301-4738.64123

**Published:** 2010

**Authors:** Robert P Rutstein

**Affiliations:** School of Optometry, University of Alabama at Birmingham, Birmingham, Alabama 35294-0010, USA

**Keywords:** Accommodative spasm, pseudomyopia, siblings

## Abstract

Accommodative spasm is a rare condition occurring in children, adolescents, and young adults. A familial tendency for this binocular vision disorder has not been reported. I describe accommodative spasm occurring in a brother and sister. Both children presented on the same day with complaints of headaches and blurred vision. Treatment included cycloplegia drops and bifocals. Siblings of patients having accommodative spasm should receive a detailed eye exam with emphasis on recognition of accommodative spasm.

Accommodative spasm (AS) is an involuntary condition when there is greater than normal accommodative response than accommodative stimulus.[1] It may begin suddenly, is more likely to be bilateral, is constant or intermittent, occurs at distance and/or near, is frequently associated with pupillary miosis and convergence spasm, disappears with cycloplegia, and may resolve spontaneously.[1] A lead of accommodation is seen with dynamic retinoscopy. With AS, hyperopes may appear less hyperopic, emmetropes may appear myopic, and myopes may appear more myopic. AS can be caused by head trauma, emotional problems, and other causes.[2,3] AS can also be intentionally induced. Diagnosed mostly in children, adolescents, and young adults, AS is rare and occurs in less than 3% of patients with accommodative disorders.[4] It occurs sporadically and familial cases have not been reported. This presentation documents AS occurring in siblings.

## Case Reports

### Case 1

**Figure 1:**
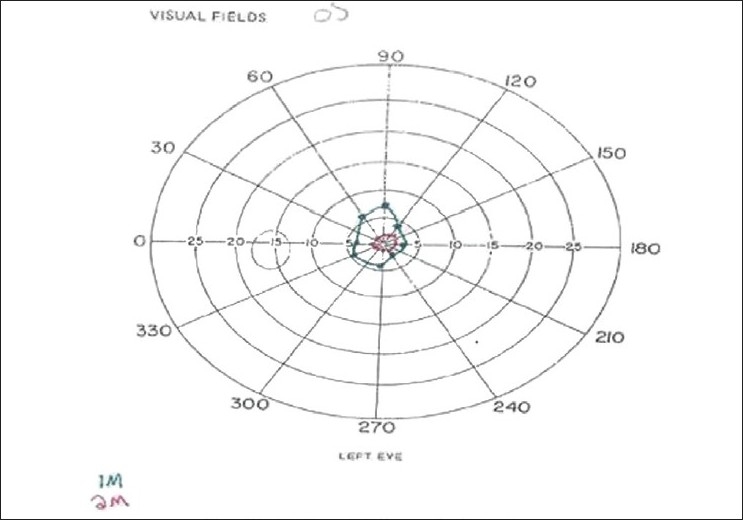
Case 1. Left visual field as measured with tangent screen is constricted and the same size at 1 and 2 meter test distances

A 13-year-old Hispanic girl was examined on January 29, 2008 with complaints of severe headaches interfering with her daily activities and blurred vision. Past ocular history was significant for lost glasses for slight myopia which were prescribed by another practitioner. The patient denied any head trauma and was not taking any medication. Presenting visual acuities were 20/400 in each eye. Orthophoria existed at distance, and 2 prism diopters (PD) esophoria at near. Stereopsis was 100 sec of arc. Versions were full as were pupillary responses to light and accommodation. There was no convergence spasm. Dynamic retinoscopy was unstable and could not be quantified. Her refractive error by retinoscopy was -8.00 diopter sphere (DS) – 1.00 Dcylinder (Dcyl) × 162 in the right eye and -4.25 DS – 0.50Dcyl × 108 in the left eye. With 1% cyclogyl, her refraction was -1.00 DS in the right eye and -0.25 DS -0.25 Dcyl × 180 in the left eye which improved visual acuities to 20/80 in each eye. Dilated ophthalmoscopy revealed normal findings. Treatment consisted of prescribing the cycloplegic refraction with a ± 2.50 DS bifocal. On March 4, the patient still had blurred vision at distance with the glasses but reported clear vision at near. Visual acuities were 20/70 in each eye. Repeat cycloplegic refraction was similar to that performed on the first visit. Cyclogyl 1% twice daily was added to the treatment. One month later, the patient reported stinging on instilling the medication and thus used it only one day since the last visit. Visual acuities were 20/80 in each eye. Retinoscopy was -11.75DS -0.25 Dcyl × 117 in the right eye and -11.25DS – 0.25Dcyl × 144 in the left eye. Visual field testing with the tangent screen revealed constricted and tubular fields [Fig. 1]. Treatment was changed to one drop atropine 1% in both eyes on weekends. Three weeks later, the patient reported elimination of all visual symptoms. Visual acuities were 20/20 in each eye. Treatment was changed to atropine 1% one day per week. On July 22, 2008, the visual acuities were 20/30 in the right eye and 20/20 in the left eye. Cycloplegic refraction was -0.50 DS in the right eye and -0.25 DS in the left eye with 20/20 in each eye. Treatment continues with atropine one day per week and bifocal lenses.

### Case 2

**Figure 2:**
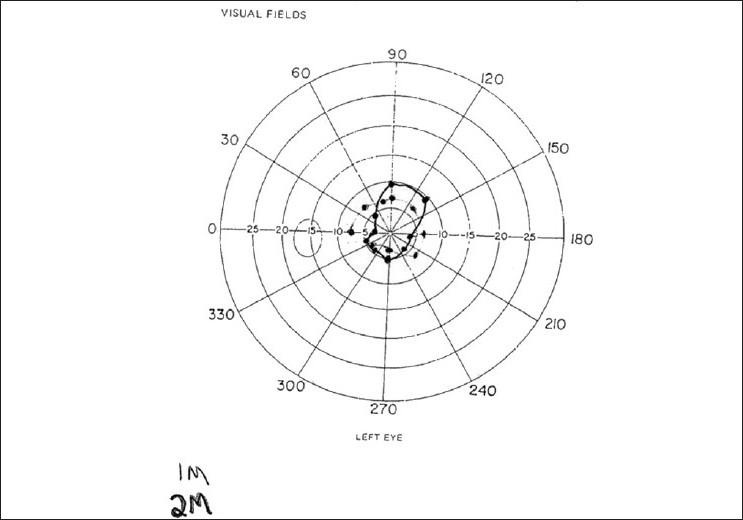
Case 2. Left visual field as measured with tangent screen is constricted and the same size at 1 and 2 meters

**Figure 3:**
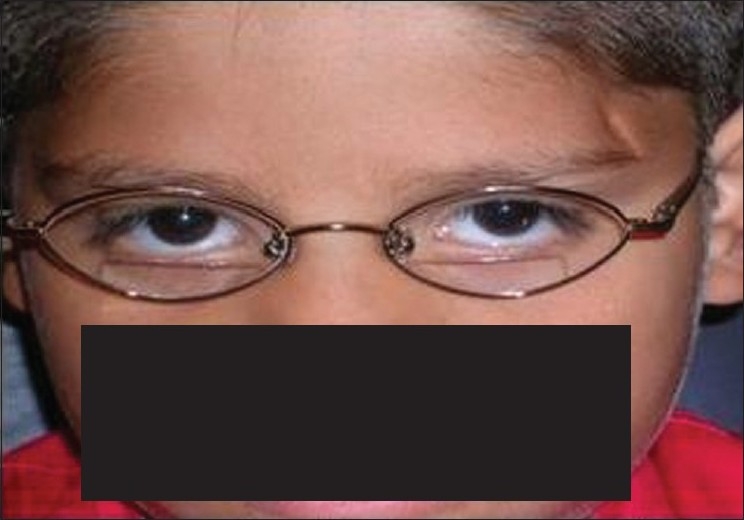
Case 2. Accommodative spasm is being treated using bifocals and atropine drops

The 10-year-old brother was examined on the same day. Similar to his sister, he was suffering from severe headaches and blurred vision. The headaches were worse when reading. This was the child's first eye examination. There was no history of either head trauma or medication. Visual acuities were 20/40 in the right eye and 20/200 in the left eye. Orthophoria existed at distance and near. With the Randot stereo test, 100 sec of arc was measured. Refraction was variable and revealed -2.50DS – 0.50 Dcyl × 90 in the right eye and -3.00 DS in the left eye. With cyclogyl 1%, the refraction was ±0.75 DS in both eyes which improved visual acuity to 20/25 in each eye. All ocular health examination including color vision was normal. Treatment consisted of the cycloplegic refraction findings and a ±2.50 DS addition for near. Compliance with treatment was poor and the glasses were worn infrequently. Visual acuities on March 4, 2008 were 20/50 in right eye and 20/60 in left eye. Cycloplegic refraction was consistent with the prior examination. Added treatment included cyclogy l 1% twice daily in each eye. At the visit on April 1, 2008, the patient had only used the drops once because of its sting. Visual acuities were 20/150 in each eye. Dynamic retinoscopy showed a large lead of accommodation. Refraction was -2.25 DS – 0.50 Dcyl × 82 in right eye and -5.50 DS– 0.50 Dcyl × 13 in left eye. Visual fields with the tangent screen were constricted and tubular [Fig. 2]. Treatment was changed to atropine 1% on weekends. On June 3, 2008, compliance with treatment was improved [Fig. 3]. Visual acuity was 20/25 in right eye and 20/30 in left eye. At the final visit on July 22, visual acuity was 20/40 in each eye. With cycloplogic refraction of ±0.75DS in both eyes, visual acuity was 20/20 in right eye and 20/30 in left eye. Treatment continues with atropine 1% one day per week and bifocal lenses.

## Discussion

To the best of this author's knowledge, these case reports are the first documentation of AS occurring in siblings. Both children manifested AS without pupillary miosis and convergence spasm, despite the AS measuring more than 11 D for one child (Case 1) on follow-up. The AS in these children may merely be coincidental. On questioning, an older sibling and both parents denied any history of AS. The association in theses siblings suggests a hereditary influence for AS but with no other reported family members and no prior case reports, it remains speculation.

The etiology of AS has been associated with diverse organic causes including closed head trauma,[2,3] multiple sclerosis,[5] intracranial hypertension due to a pineal cyst,[6] a blocked ventriculo-peritoneal shunt,[7] laser-assisted *in situ* keratomileusis (LASIK),[8] acute respiratory disease,[9] and certain ocular or systemic pharmacological agents.[1] AS more commonly occurs as an isolated functional entity, usually attributed to psychogenic causes. The latter seems probable for these siblings even though they seemed well adjusted and without signs of stress, anxiety or depression. The visual fields as measured with the tangent screen were constricted and nonexpanding with increasing test distances which is pathognomonic for a psychogenic cause.[10] However, both parents and patients denied problems in family or school.

Although nonorganic AS can resolve spontaneously over time, treatment is usually indicated because of the severe symptoms. Treatment for AS involves inhibiting the excessive accommodative tone by prescribing bifocals with or without cycloplegic drops. Bifocals alone were insufficient and atropine drops given on weekends were added. Presently, both patients are using atropine drops one day per week with the bifocals. The goal is to wean entirely from the drops and the bifocals and not have the AS recur.

In summary, siblings of patients having AS should receive a detailed eye examination with emphasis on recognizing AS.
